# Taxonomic review of the genus *Nycteola* Hübner (Lepidoptera, Nolidae) from Korea including potential invasive pests

**DOI:** 10.3897/BDJ.11.e114878

**Published:** 2023-12-29

**Authors:** Yeong-Bin Cha, Un-Hong Heo, Ulziijargal Bayarsaikhan, Sora Kim, Yang-Seop Bae

**Affiliations:** 1 Lab. of insect Phylogenetics & Evolution, Jeonju, Republic of Korea Lab. of insect Phylogenetics & Evolution Jeonju Republic of Korea; 2 88, Sangam-ro 79-gil, Gangdong-gu, Seoul, Republic of Korea 88, Sangam-ro 79-gil, Gangdong-gu Seoul Republic of Korea; 3 Convergence Research Center for Insect Vectors, Incheon, Republic of Korea Convergence Research Center for Insect Vectors Incheon Republic of Korea; 4 Jeonbuk National University, Jeonju, Republic of Korea Jeonbuk National University Jeonju Republic of Korea; 5 Incheon National University, Incheon, Republic of Korea Incheon National University Incheon Republic of Korea

**Keywords:** endemic species, Japan, new record, Palearctic region, synonym

## Abstract

**Background:**

The genus *Nycteola* Hübner has been mainly distributed in the Old World and usually feeds on Fagaceae and Salicaceae, but Myrtaceae and Juglandaceae have also been reported. In Korea, the number of this genus has been changed from four to three after 2007, but three or four species are listed confusingly up to now.

**New information:**

The Japanese endemic species *Nycteoladufayi* Sugi, 1982 are firstly reported for the Continents with its brief biology. Additionally, Korean fauna of nycteolid species are reviewed.

## Introduction

The genus *Nycteola* was established by Hübner (1822), with the type species, *Tortrixundulana* Hübner [1799] by Prout (1901) subsequently. This genus is mainly characterised by the male abdomen: lack of a tymbal organ and anterior apodemes at the eighth segment (Holloway 2003). Additionally, the male genitalia has several noticeable features: a broad uncus with a gnathos-like structure, tegumen broadened at the base with a bunch of hair, a very complex valva convoluted with several lobes and hair and setae, a vinculum that is very long and loop-shaped, a very distinctive axe-head-shaped saccular shield and a normally slender aedeagus, sometimes partly attached to the saccular shield (Holloway 2003). In the female genitalia, triangular papillae anales are characteristic, but the ductus bursae and corpus bursae do not show unified characteristics (Holloway 2003).

The genus is mainly distributed in the Old World, especially Western Palaearctic and Indomalayan Regions, as well as the New World, except for Polar Regions (Holloway 2003). They usually feed on Fagaceae and Salicaceae, but it has also been reported that they feed on Myrtaceae and Juglandaceae (Yamamoto and Sugi 1987, Holloway 2003, Sohn et al. 2005, Fibiger et al. 2009, Sasaki and Kishida 2011, Heo 2012, Beljaev et al. 2016, Jinbo et al. 2019, Heo 2021). This association implies that, if a new nycteolid species is reported anywhere, it could be suspected of being a forest pest.

In Korea, four species (*N.asiatica* (Krulikowski, 1904), *N.coreana* (Leech, 1900), *N.costalis* Sugi, 1959 and *N.degenerana* (Hübner, 1799)) have been recorded after Sohn et al. (2005). However, Kononenko and Han (2007) synonymised *N.costalis* Sugi with *N.coreana* (Leech) in some literature, but this change was not adapted by all sources (Paek et al. 2010, Sasaki and Kishida 2011, National Institute of Biological Resources 2019). Recently, Park and Lee (2021) adapted three *Nycteola* species lists.

Here, *N.dufayi* Sugi, 1982, which has only been distributed in Japan since its description, is reported for the first time on the Asiatic continent, specifically in Korea, along with a new host plant and brief biology. This species was originally described between Kyushu and Honshu (Tokyo) and was also reported to feed on Fagaceae in Japan (Sasaki and Kishida 2011, Jinbo et al. 2019).

As a result of this study, four species of Korean Nycteola have been confirmed. *Nycteolacostalis* Sugi is a junior synonym of *N.coreana* (Leech) and *N.dufayi* Sugi is newly reported from both Korea and the continent.

## Materials and methods

Specimens examined are preserved in the collections of the Incheon National University, South Korea (INU) and the Korean National Arboretum, Pocheon, Korea (KNAE, HEO). Genitalia were dissected and examined using a Leica EZ4 stereomicroscope. Images of adults were taken by a Tucsen Dhyana 400DC digital camera attached to a Leica S6D stereomicroscope, with dome illuminator Leica LED5000 HDI. Genitalia photograph were taken using a Tucsen Dhyana 400DC digital camera mounted on a Leica S8AP0 stereomicroscope.

Further abbreviations:

- CB: Chungcheongbuk-do Province.
- GG: Gyeonggi-do Province.
- GW: Gangwon-do Province.
- HEO: Un-Hong Heo’s private collection.
- JB: Jeollabuk-do Province.
- JJ: Jeju-do Island.
- JN: Jeollanam-do Province.
- NHMUK: The Natural History Museum, London, United Kingdom
- NIAES: National Institute for Agro-Enviromental Sciences, Ibaraki, Japan.
- ZI: Zoological Institute, Academy of Sciences of the U.S.S.R., Saint Petersburg (Leningrad), Russia.

## Taxon treatments

### Nycteola

Hübner, 1822

90BCFDA1-B730-5110-8446-A0558210A452

Nycteola Hübner, 1822: 60, 66. Type species: *Tortrixundulana* Hübner, [1799] by subsequent designation by Prout (1901): 183.
Sarrothripus Curtis, 1824 - Curtis 1824: 29. Type species: *Tortrixdegenerana* Hübner, 1799.
Axia Hübner, 1825 - Hübner 1825: 395. Type species: *Phalaenarevayana* Scopoli, 1772.
Symitha Walker, 1866 - Walker 1866: 1731. Type species: *Symithanolalella* Walker, 1866.
Subrita Walker, 1866 - Walker 1866: 1744. Type species: *Subritabilineatella* Walker, 1866.
Sarotricha Meyrick, 1888 - Meyrick 1888: 924 (emend.). Type species: *Sarotrichaexophila* Meyrick, 1888.
Icasma Turner, 1902 - Turner 1902: 90. Type species: *Icasmaminutum* Turner, 1902.
Dufayella Capuse, 1972 - Capuse 1972: 89. Type species: Sarrothripusrevayanavar.asiatica Krulikovsky, 1904.
Nycteola Tortrix undulana Hübner, 1799

#### Diagnosis

This genus is characterised by its rectangular forewing, with a variety of markings. Their venation is typical trifine (M3 and CuA1 stalked in the hindwing). The tymbal of abdomen absent, but the 8^th^ segment is noticeable. The male genitalia has broad uncus, basally expanded tegumen, long loop like vinculum and the very distinct axe-head-shaped saccular shield, with very complexed valva. The female genitalia do not have characteristic features, but usually have triangular to acute ovipostior lobes (genus characteristics after Holloway (2003)).

### Nycteola asiatica

(Krulikovsky, 1904)

A9B54886-8F89-5F2B-8B89-0E380093BDDE

Sarrothripus revayana var. asiatica Krulikovsky, 1904 - Krulikovsky 1904: 91. Type locality: [USSR]: [Turkmen SSR], Transcaspicae, Aschabad. Types in coll. ZI.
Sarrothripus populana Patocka, 1953 - Patocka 1953: 77. Type locality: South Slovakia.
Sarrothripus hungarica Kovács, 1954 - Kovács 1954: 306. Type locality: Hungary: Batorliget.
Nycteola pseudasiatica Sugi, 1959 - Sugi 1959: 277. Type locality: Japan: Tokyo.
Nycteola revayana (Scopoli, 1772) - Kim et al. 1982 - Kim et al. 1982: 415 (misidentification)
Nycteola asiatica : Pak, 1959 - Pak 1959: 38; Inoue 1982: 377, 794; Poole 1989: 704; ESK and KSAE 1994: 374; Kononenko et al. 1998: 140; Kononenko and Han 2007: 72; Paek et al. 2010: 304; Sasaki and Kishida 2011: 183; Heo 2012: 358; Kim et al. 2016: 141; National Institute of Biological Resources 2019: 605; Park and Lee 2021: 615.

#### Materials

- **Type status:** Other material. **Occurrence:** catalogNumber: INU-12088; recordedBy: Lee, Ahn, Oh & Lee; individualCount: 1; sex: 1 male; lifeStage: adult; occurrenceID: 80630AF7-7BB1-55CE-BCBC-18965A9AA88C; **Taxon:** class: Insecta; order: Lepidoptera; family: Nolidae; genus: Nycteola; specificEpithet: *asiatica*; taxonRank: species; **Location:** country: Korea; stateProvince: CB; locality: Oksam S.A.; **Identification:** identifiedBy: Y.B. Cha; dateIdentified: 2022; **Event:** eventTime: 02-05-1997; **Record Level:** language: en; collectionCode: Insects; basisOfRecord: PreservedSpecimen
- **Type status:** Other material. **Occurrence:** catalogNumber: INU-12081, -12082, -12083, -12084, -12085; recordedBy: Kwon Y.D. & Lee H.K.; individualCount: 5; sex: 2 males, 3 females; lifeStage: adult; occurrenceID: 8A95D123-08CF-5946-8D39-C50260881FF3; **Taxon:** class: Insecta; order: Lepidoptera; family: Nolidae; genus: Nycteola; specificEpithet: *asiatica*; taxonRank: species; **Location:** country: Korea; stateProvince: GG; locality: Suchung-dong, Osan-si; **Identification:** identifiedBy: Y.B. Cha; dateIdentified: 2022; **Event:** eventTime: 21-04-1998; **Record Level:** language: en; collectionCode: Insects; basisOfRecord: PreservedSpecimen
- **Type status:** Other material. **Occurrence:** catalogNumber: INU-12086; recordedBy: Lee & Kim; individualCount: 1; sex: 1 male; lifeStage: adult; occurrenceID: 51CF2F05-5594-5353-87AB-70C0FBB45784; **Taxon:** class: Insecta; order: Lepidoptera; family: Nolidae; genus: Nycteola; specificEpithet: *asiatica*; taxonRank: species; **Location:** country: Korea; stateProvince: GG; locality: Mt. Yongmun, Yangpyeong-gun; **Identification:** identifiedBy: Y.B. Cha; dateIdentified: 2022; **Event:** eventTime: 28-07-2000; **Record Level:** language: en; collectionCode: Insects; basisOfRecord: PreservedSpecimen
- **Type status:** Other material. **Occurrence:** catalogNumber: INU-12087; recordedBy: Bae et al.; individualCount: 1; sex: 1 female; lifeStage: adult; occurrenceID: 230CF269-1B13-5EEC-8B49-1D0A325E2175; **Taxon:** class: Insecta; order: Lepidoptera; family: Nolidae; genus: Nycteola; specificEpithet: *asiatica*; taxonRank: species; **Location:** country: Korea; stateProvince: GG; locality: Yangsu-ri, Yangpyeong-gun; **Identification:** identifiedBy: Y.B. Cha; dateIdentified: 2022; **Event:** eventTime: 29-08-2004; **Record Level:** language: en; collectionCode: Insects; basisOfRecord: PreservedSpecimen
- **Type status:** Other material. **Occurrence:** catalogNumber: INU-11843, -11845, -11846; recordedBy: Park et al.; individualCount: 3; sex: 1male, 2 females; lifeStage: adult; occurrenceID: 09479FC7-DFD8-5EFF-81EB-D1B2864E98E6; **Taxon:** class: Insecta; order: Lepidoptera; family: Nolidae; genus: Nycteola; specificEpithet: *asiatica*; taxonRank: species; **Location:** country: Korea; stateProvince: GW; locality: Gangwon National Univ., Chuncheon; **Identification:** identifiedBy: Y.B. Cha; dateIdentified: 2022; **Event:** eventTime: 26-04-2003; **Record Level:** language: en; collectionCode: Insects; basisOfRecord: PreservedSpecimen
- **Type status:** Other material. **Occurrence:** catalogNumber: INU-11847, -11920, -11921, -11922, -11923, -11924, -11925; recordedBy: Park et al.; individualCount: 7; sex: 2 male, 5 females; lifeStage: adult; occurrenceID: EA7AC41C-A3A1-504E-9120-F548DCA443DB; **Taxon:** class: Insecta; order: Lepidoptera; family: Nolidae; genus: Nycteola; specificEpithet: *asiatica*; taxonRank: species; **Location:** country: Korea; stateProvince: GW; locality: Gangwon National Univ., Chuncheon; **Identification:** identifiedBy: Y.B. Cha; dateIdentified: 2022; **Event:** eventTime: 01-05-2003; **Record Level:** language: en; collectionCode: Insects; basisOfRecord: PreservedSpecimen
- **Type status:** Other material. **Occurrence:** catalogNumber: INU-12089; recordedBy: Bae et al.; individualCount: 1; sex: 1 male; lifeStage: adult; occurrenceID: 9B9E5732-09D5-5829-A8A3-87A4695880EC; **Taxon:** class: Insecta; order: Lepidoptera; family: Nolidae; genus: Nycteola; specificEpithet: *asiatica*; taxonRank: species; **Location:** country: Korea; stateProvince: JB; locality: Mt. Daedunsan, Wanju-gun; **Identification:** identifiedBy: Y.B. Cha; dateIdentified: 2022; **Event:** eventTime: 13-05-2000; **Record Level:** language: en; collectionCode: Insects; basisOfRecord: PreservedSpecimen
- **Type status:** Other material. **Occurrence:** catalogNumber: INU-11610; recordedBy: Bae et al.; individualCount: 1; sex: 1 female; lifeStage: adult; occurrenceID: 4733AA7F-9C35-5B95-9EB0-2A3942B2E604; **Taxon:** class: Insecta; order: Lepidoptera; family: Nolidae; genus: Nycteola; specificEpithet: *asiatica*; taxonRank: species; **Location:** country: Korea; stateProvince: JB; locality: Temp. Naesosa, Buan-gun; **Identification:** identifiedBy: Y.B. Cha; dateIdentified: 2022; **Event:** eventTime: 02-05-2004; eventRemarks: Host plant: *Salixkoreensis*; **Record Level:** language: en; collectionCode: Insects; basisOfRecord: PreservedSpecimen
- **Type status:** Other material. **Occurrence:** catalogNumber: INU-11841; recordedBy: Byun B.K.; individualCount: 1; sex: 1 female; lifeStage: adult; otherCatalogNumbers: KNAE25608; occurrenceID: 7F3D4C32-BF93-56B9-B310-379F9BA54D1A; **Taxon:** class: Insecta; order: Lepidoptera; family: Nolidae; genus: Nycteola; specificEpithet: *asiatica*; taxonRank: species; **Location:** country: Korea; stateProvince: GG; locality: Exotic trees garden, Gwangneung; verbatimCoordinates: 37°45'23.65"N 127°09'45.96"E; **Identification:** identifiedBy: Y.B. Cha; dateIdentified: 2022; **Event:** eventTime: 18-04-2005; **Record Level:** language: en; collectionCode: Insects; basisOfRecord: PreservedSpecimen
- **Type status:** Other material. **Occurrence:** catalogNumber: INU-9191; recordedBy: Park S.Y., Lee B.W., Kim S.R., Kwon D.H.; individualCount: 1; sex: 1 female; lifeStage: adult; otherCatalogNumbers: KNAE198091; occurrenceID: 3903DF46-8B3F-5248-A140-29CD31A5AEC7; **Taxon:** class: Insecta; order: Lepidoptera; family: Nolidae; genus: Nycteola; specificEpithet: *asiatica*; taxonRank: species; **Location:** country: Korea; stateProvince: GG; locality: Lake Yuklim, Gwangneung; verbatimCoordinates: 37°44'54.73"N 127°04'50.09"E; **Identification:** identifiedBy: Y.B. Cha; dateIdentified: 2022; **Event:** eventTime: 10-06-2008; **Record Level:** language: en; collectionCode: Insects; basisOfRecord: PreservedSpecimen

#### Description

**Adults.** (Fig. 1a, b) Length of forewing 10–12 mm in both sexes, wingspan 22–27 mm. Antenna filiform in both sexes. Head and thorax grey. Ground colour of forewing grey; black sub-basal line indistinct; costal half of double antemedial line bent, dorsal half zigzagged; medial fascia ochrous, black or indistinct, but noticeable costally; a reddish-ochrous discal dot near postmedial line; postmedial line doubled, unclear, somewhat zigzagged, wavy; subterminal line dotted, angled twice. Ground colour of hindwing pale ivory, darkening towards termen. Abdomen grey.

**Male genitalia.** (Fig. 2a, c, e) Uncus sclerotised, truncate, apically rounded, with subscaphium. Tegumen narrow, peniculus trapezoidally expanded. Transtilla weakly sclerotised. Valva trapezoid; costal margin straight with a finger-shaped process; apical lobe hairy; harpe-like process towards apex, apically clothed with long setae; ventro-distal lobe complex and convolute. Saccular shield elongated spatulate, partly fused to aedeagus. Vinculum rather elongated U-shaped. Aedeagus short, stout, with a band of cornuti and a rather large conspicuous cornutus. Eighth tergite costally diagonal wing-shaped plate with two short clavate processes anteriorly, postero-laterally weakly sclerotised; 8^th^ sternite with anteriorly bent H-shaped plate.

**Female genitalia.** (Fig. 3a) Apophyses anteriores half-length of apophyses posteriores. Ostium bursae membranous. Ductus bursae membranous, intertwined with a strong sclerite, with cervix bursae. Corpus bursae membranous, weakly wrinkled, strongly curved. Appendix bursae absent.

#### Diagnosis

This species can be distinguished from other congeners by its a reddish discal dot on the forewing and pale ground colour of hindwing in the adult. In the male genitalia, only *asiatica* have aedeagus with a conspicuous spinous cornutus and a row of cornuti fields. In the female genitalia, its large convolute ductus bursae can identify it from others.

#### Distribution

Korea, Japan, China, Russian Far East, Nepal, N India, Afghanistan, Turkmenistan, Central Asia, Iran, the Middle East, Turkey, Caucasus, European part of Russia, Europe (Kononenko et al. 1998, Fibiger et al. 2009, Sasaki and Kishida 2011, Beljaev et al. 2016).

#### Ecology

**Host plant**: *Salixeriocarpa*, *S.koreensis*, *S.koriyanagi* (Salicaceae) (Heo 2012, Jinbo et al. 2019).

#### Notes

While it is noted that Pak (1959) initially reported the species in Korea, he recorded it as a previously reported species. Nonetheless, based on our findings and current available literature, we believe this to be the first official record of the species in Korea. Kim et al. (1982) only reported *N.revayana* (Scopoli), but this species has not been listed up to now.

### Nycteola coreana

(Leech, 1900)

699A7D4B-9FE8-5984-BDBA-B61F0E6DDA11

Sarrothripus coreana Leech, 1900 - Leech 1900: 518. Type locality: Korea: Gensan. Holotype: male, in coll. NHMUK.
Nycteola costalis Sugi, 1959 - Sugi 1959: 278. Type locality: Japan: Tokyo: Takao-san.
Nycteola costalis : Poole, 1989 - Poole 1989: 704; Sohn et al. (2005): 220; Paek et al. 2010: 304; Kim et al. (2016): 141; National Institute of Biological Resources 2019: 605.
Nycteola coreana : Pak, 1959 - Pak (1959): 38; Kim et al. 1982: 415; Poole 1989: 704; ESK and KSAE 1994: 374; Kononenko and Han (2007): 72; Paek et al. 2010: 304; Kim et al. (2016): 141; National Institute of Biological Resources 2019: 605; Park and Lee 2021: 615.

#### Materials

- **Type status:** Other material. **Occurrence:** catalogNumber: INU-12096; recordedBy: Lee, Kim & Song; individualCount: 1; sex: 1 male; occurrenceID: BE5B9666-A3C2-5C63-BC7E-F02CDC5D5386; **Taxon:** class: Insecta; order: Lepidoptera; family: Nolidae; genus: Nycteola; specificEpithet: *coreana*; taxonRank: species; **Location:** country: Korea; stateProvince: JJ; locality: Seongpanak, Jeju-si; **Identification:** identifiedBy: Y.B. Cha; dateIdentified: 2022; **Event:** eventDate: 26-07-2003; **Record Level:** language: en; collectionCode: Insects; basisOfRecord: PreservedSpecimen
- **Type status:** Other material. **Occurrence:** catalogNumber: INU-11953; recordedBy: Bae Y.S., Park B.S., Na S.M. Lee D.J.; individualCount: 1; sex: 1 female; occurrenceID: 4A0FB28B-DF31-529C-BB9A-785FC1683202; **Taxon:** class: Insecta; order: Lepidoptera; family: Nolidae; genus: Nycteola; specificEpithet: *coreana*; taxonRank: species; **Location:** country: Japan; stateProvince: Nagasaki; locality: Ayumodoshi(1), Tsushima; verbatimCoordinates: 34°09'12.96"N 129°12'58.48"E; **Identification:** identifiedBy: Y.B. Cha; dateIdentified: 2022; **Event:** eventDate: 22-06-2015; **Record Level:** language: en; collectionCode: Insects; basisOfRecord: PreservedSpecimen

#### Description

**Adults.** (Fig. 1c, d) Length of forewing 9 mm in male, 11 mm in female, wingspan 20–24 mm (Sohn et al. 2005). Antenna filiform in both sexes. Head and thorax grey. Ground colour of forewing grey; longitudinal basal fascia black; black medial line undulate, wavy; medial fascia black, triangular; a blackish streak under medial fascia; postmedial line undulate, wavy; subterminal line somewhat indistinct, undulate, wavy. Ground colour of hindwing dark grey. Abdomen grey.

**Male genitalia.** (Fig. 2b, d, f) Uncus stout, rounded end, with subscaphium. Tegumen narrow, broadened basally. Valva triangular; costal margin almost straight with a bent spinous process; apex lobe hairy, supported by harpe-like process towards apex; tornal lobe hairy; ventral margin somewhat wavier than costal margin. Saccular shield anchor-shaped, partly fused to aedeagus. Vinculum rectangular. Aedeagus long, slender, with a double-bladed saw-shaped cornutus. Eighth tergite costally horizontal wing-shaped plate with two long clavate processes anteriorly; postero-laterally weakly sclerotised, with a membranous pouch posteriorly; 8^th^ sternite with butterfly-shaped plate.

**Female genitalia.** (Fig. 3b) Apophyses anteriores somewhat longer than apophyses posterior. Ostium bursae membranous, narrow to ductus bursae. Ductus bursae membranous, with weakly sclerotised area in 1/3 from posterior; appendix bursae based on 2/3 of ductus bursae. Corpus bursae ovate.

#### Diagnosis

This species can be distinguished from other congeners by its a conspicuous triangular black costal patch with a tornal black dot on the forewing in the adult. In the male genitalia, only the *coreana* has a significantly slender aedeagus with a saw-shaped cornutus and a row of cornuti fields. In the female genitalia, its noticeable appendix bursae at ductus bursae can identify it from others.

#### Distribution

Korea, Japan (Sasaki and Kishida 2011).

#### Ecology

**Host plant**: *Quercus* spp., *Q. acuta, Q*. *glauca* (Fagaceae) (Yamamoto and Sugi 1987, Sohn et al. 2005, Heo 2021).

#### Notes

This species was firstly recorded from the Korean Peninsula by Leech (1900). Sohn et al. (2005) recorded *N.costalis* Sugi from Wando Is. from South Korea, but it was synonymised by Kononenko and Han (2007) as *N.coreana*. According to our findings in this study, while Poole (1989) was the first to describe *N.coreana* as a new combination from *Sarrothripus* to *Nycteola*, an earlier combination was discovered in the bibliography of Pak (1959).

### Nycteola degenerana

(Hübner, 1799)

BB2C01FA-BB33-5868-80E8-93718E9839D0

Tortrix degenerana Hübner, 1799 - Hübner 1799: pl.2, fig. 8. Type locality: Europe.
Nycteola degenerana hesperica Dufay, 1958 - Dufay 1958: 112. Type locality: [France]: Pyrenees: St.-Pierra d'Irrube.
Nycteola degenerana eurasiatica Dufay, 1961 - Dufay 1961: 434. Type locality: [Russia]: Moscow.
Nycteola degenerana eurasiatica : Ronkay and Park, 1993 - Ronkay and Park (1993): 65.
Nycteola degenerana : Poole (1989): 704; Kononenko et al. (1998): 140; Kononenko and Han (2007): 72; Paek et al. 2010: 304; Sasaki and Kishida (2011): 183; Beljaev et al. 2016: 404; Kim et al. (2016): 141; National Institute of Biological Resources 2019: 605; Park and Lee 2021: 615.

#### Materials

- **Type status:** Other material. **Occurrence:** catalogNumber: INU-11609; recordedBy: Bae, Paek & Lee; individualCount: 1; sex: 1 male; lifeStage: adult; occurrenceID: 67A18872-ECA7-58D9-B624-A2F8866A5EE0; **Taxon:** class: Insecta; order: Lepidoptera; family: Nolidae; genus: Nycteola; specificEpithet: *degenerana*; taxonRank: species; **Location:** country: Korea; stateProvince: GW; locality: Mt. Kyebang, Hongcheon; **Identification:** identifiedBy: Y.B. Cha; dateIdentified: 2022; **Event:** eventDate: 26-05-1996; **Record Level:** language: en; collectionCode: Insects; basisOfRecord: PreservedSpecimen
- **Type status:** Other material. **Occurrence:** catalogNumber: INU-11844; recordedBy: Park et al.; individualCount: 1; sex: 1 female; lifeStage: adult; occurrenceID: 48D13D20-EBB4-5E69-97E3-3D9C012EF9D8; **Taxon:** class: Insecta; order: Lepidoptera; family: Nolidae; genus: Nycteola; specificEpithet: *degenerana*; taxonRank: species; **Location:** country: Korea; stateProvince: GW; locality: Gangwon National Univ., Chuncheon; **Identification:** identifiedBy: Y.B. Cha; dateIdentified: 2022; **Event:** eventDate: 26-04-2003; **Record Level:** language: en; collectionCode: Insect; basisOfRecord: PreservedSpecimen

#### Description

**Adults.** (Fig. 4a, b) Length of forewing 11–12 mm in both sexes, wingspan 25 mm. Antenna filiform in both sexes. Head and thorax greenish-grey. Ground colour of forewing greenish-grey; basal band angled, fuscous; sub-basal line parallel to basal line; antemedial line indistinct; medial line and postmedial line doubled, wavy; subterminal line undulated. Ground colour of hindwing pale grey, darkening towards termen. Abdomen pale grey.

**Male genitalia.** (Fig. 5a, c, e) Uncus sclerotised, triangular, rounded end, with subscaphium. Tegumen narrow; peniculus round trapezoidally expanded. Transtilla weakly sclerotised. Valva costal margin bent with a short spinous process; apex lobe hairy; harpe-like process towards apex, apically clothed with long hair; ventro-distal lobe complex and convolute. Sacculus sclerotised. Saccular shield elongated spatulate, axe-head-shaped; partly fused to aedeagus. Vinculum rather elongated U-shaped. Aedeagus long, slender, broadened in apical 1/3, carina process snail-shell-shaped. Eighth tergite costally diagonal wing-shaped plate with two short clavate processes anteriorly, with postero-laterally weakly sclerotized; 8^th^ sternite anteriorly curved H-shaped.

**Female genitalia.** (Fig. 3c) Apophyses anteriores somewhat shorter than apophyses posterior. Ostium bursae membranous. Ductus bursae short, sclerotised, stout, with cervix bursae. Corpus bursae somewhat peanut-shaped. Appendix bursae absent.

#### Diagnosis

This species can be distinguished from other congeners by its fuscous green forewing colour in the adult. In the male genitalia, only *degenerana* have aedeagus with a conspicuous snail-shell-shaped carina precess. In the female genitalia, its well sclerotised ductus bursae with somewhat peanut-shaped corpus bursae can identify it from others.

#### Distribution

Korea, Spain, France, Switzerland, Italy, Slovenia, Austria, Czech, Germany, Poland, Slovakia, Rumania, Ukraine, Belarus, Lithuania, Latvia, Estonia, Norway, Sweden, Finland, Russia, Central Asia, northern China, Mongolia (Beljaev et al. 2016, Joshi et al. 2021).

#### Ecology

**Hostplant**: *Salixcaprea* (Salicaceae) (Fibiger et al. 2009).

#### Notes

This species was firstly recorded from the Korean Peninsula by Ronkay and Park (1993) from North Korea as *Nycteoladegeneranaeurasiatica* Dufay and South Korea by Kononenko and Han (2007). According to Beljaev et al. (2016), the species is distributed in India; however, it was not listed in the study conducted by Joshi et al. (2021). Therefore, we do not accept the distribution of the species in India.

### Nycteola dufayi

Sugi, 1982

D9255002-20F4-5C71-AC34-F3FA1D7E2BE6

Nycteola dufayi Sugi in Inoue, 1982 - Inoue 1982: 794. Type locality: Japan: Honshu: Tokio [Tokyo]: Takaosan. Holotype: male, in coll. NIAES.
Nycteola dufayi : Poole, 1989 - Poole 1989:704; Sasaki and Kishida, 2011 - Sasaki and Kishida 2011: 183.

#### Materials

- **Type status:** Other material. **Occurrence:** catalogNumber: INU-11971, -11972; recordedBy: Heo W.H.; individualCount: 2; sex: 2 males; occurrenceID: 09064A2D-F535-5108-844E-B823FDA35776; **Taxon:** class: Insecta; order: Lepidoptera; family: Nolidae; genus: Nycteola; specificEpithet: *dufayi*; taxonRank: species; **Location:** country: Korea; stateProvince: JN; locality: Mt. Wanguisan, Suncheon-si; **Identification:** identifiedBy: Y.B. Cha; dateIdentified: 2022; **Event:** eventDate: 02-08-2016; eventRemarks: Host plant: *Quercusserrata*, emerged 13. VIII. 2016; **Record Level:** rightsHolder: Un-Hong Heo; collectionCode: Insects; basisOfRecord: PreservedSpecimen
- **Type status:** Other material. **Occurrence:** catalogNumber: INU-11973; recordedBy: Heo W.H.; individualCount: 1; sex: 1 female; lifeStage: adult; occurrenceID: E0D66A07-C0CB-5C62-8EAA-5AF871C62F27; **Taxon:** class: Insecta; order: Lepidoptera; family: Nolidae; genus: Nycteola; specificEpithet: *dufayi*; taxonRank: species; **Location:** country: Korea; stateProvince: JN; locality: Mt. Wanguisan, Suncheon-si; **Identification:** identifiedBy: Y.B. Cha; dateIdentified: 2022; **Event:** eventDate: 02-08-2016; eventRemarks: Host plant: *Quercusserrata*, emerged 11. VIII. 2016; **Record Level:** rightsHolder: Un-Hong Heo; collectionCode: Insects; basisOfRecord: PreservedSpecimen

#### Description

**Adults.** (Fig. 4c, d) Length of forewing 9–10 mm in both sexes, wingspan 19–21 mm. Antenna filiform in both sexes. Head and thorax brownish-grey, with a black strigular on tegula. Ground colour of forewing brownish-grey; basal and sub-basal lines indistinct fuscous grey; antemedial line irregulary dentate; medial area bright grey with a small, dark grey discal dot and a large black dot near dorsal margin; medial line wavy, dentate; postmedial line dotted, wavy; teminal margin triangular dotted on each vein. Ground colour of hindwing fuscous grey. Abdomen fuscous grey.

**Male genitalia.** (Fig. 5b, d, f) Uncus sclerotised, rounded end, with subscaphium. Tegumen narrow; peniculus roundly expanded. Transtilla weakly sclerotised. Valva narrow; costal margin bent; apex lobe with hairy; subapical lobe with series of distinct spines; harpe-like process towards apex, apically clothed with long, stout setae; ventro-distal lobe complex and convolute. Sacculus sclerotised. Saccular shield elongated spatulate, somewhat axe-head-shaped; partly fused to aedeagus. Vinculum U-shaped. Aedeagus long, slender, slightly bent with spinule vesica; carina process porrect. Eighth tergite costally horizontal wing-shaped plate with two long clavate processes anteriorly, postero-laterally weakly sclerotised; 8^th^ sternite anteriorly angled H-shaped.

**Female genitalia.** (Fig. 3d) Apophyses anteriores somewhat shorter than apophyses posterior. Ostium bursae membranous, narrow. Ductus bursae sclerotised posteriorly and membranous anteriorly. Corpus bursae pyriform. Appendix bursae absent.

#### Diagnosis

This species can be distinguished from other congeners by its a small reddish discal dot with a large black dot near dorsal margin on the forewing in the adult. In the male genitalia, only *dufay* have distinctive aedeagus with spinule vesica. In the female genitalia, its long slender ductus bursae can identify it from others.

#### Distribution

Korea (new record), Japan (Sasaki and Kishida 2011).

#### Ecology

**Host plant**: *Quercus glauca, Q*. *gilva, Q*. *salicina*, *Castanopsissieboldiisieboldii* (Fagaceae) (Jinbo et al. 2019). *Quercusserrata* (Fagaceae) (new record).

#### Notes

This species has been only recorded from Japan, but newly recorded from the continent herein with a new host plant. Here, we show the brief biology of this species in Fig. 6.

## Identification Keys

### Key of the genus *Nycteola* Hübner in Korea

**Table:** 

1	Adult hindwing ground colour creamy and dark grey veins noticeable	*N.asiatica*
–	Adult hindwing ground colour fuscous and dark grey veins unnoticeable	2
2	Adult forewing with a distinct triangular patch on costal margin	*N.coreana*
–	Adult forewing without triangular patch	3
3	Adult forewing with a large black dot near tornal margin and hindwing dark grey	*N.dufayi*
–	Adult forewing with a small black dot near tornal margin and hindwing pale grey	*N.degenerana*

## Discussion

The Korean *Nycteola* group was discussed. This group is mainly distributed in the Old World and is sometimes widely spread (Holloway 2003). Amongst them, only three species are recorded in Korea, and four in Japan (Sasaki and Kishida 2011). For almost 40 years, the *Nycteola* fauna of both countries has not changed, but this report highlights the discovery of the Japanese endemic species *N.dufayi* Sugi.

This report is worth considering in the context of global warming and invasive species. According to Sasaki and Kishida (2011), this species has only been recorded in Honshu, Shikoku and Kyushu and it was suspected that the Kanto area is the northern limit. These regions are located further south and are normally warmer than the northern area. During this study, three larvae were found on Mt. Wanguisan, at the centre of Suncheon City and were reared and hatched (Fig. 6). Additionally, as it is located to the southwest of Japan, it may not be affected by the westerly airstream, but perhaps only by typhoons. Therefore, the Japanese endemic species *N.dufayi* might become invasive outside of Japan due to global warming, allowing it to inhabit more northern areas. In Japan as well, *N.dufayi* Sugi’s distribution may shift northwards. Therefore, it could serve as an indicator of climate change.

## Supplementary Material

XML Treatment for Nycteola

XML Treatment for Nycteola asiatica

XML Treatment for Nycteola coreana

XML Treatment for Nycteola degenerana

XML Treatment for Nycteola dufayi

## Figures and Tables

**Figure 1a.**
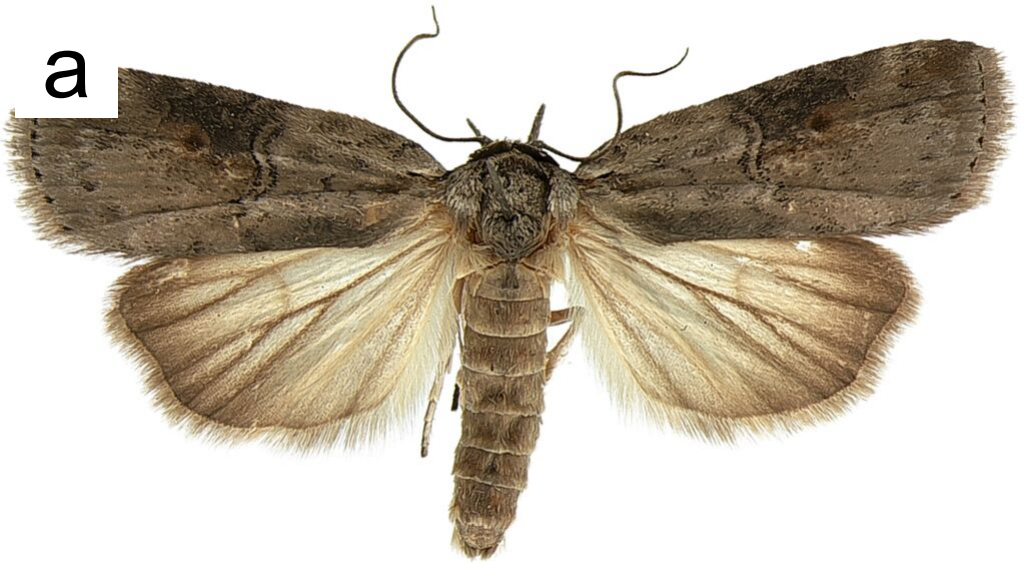
*N.asiatica*, male;

**Figure 1b.**
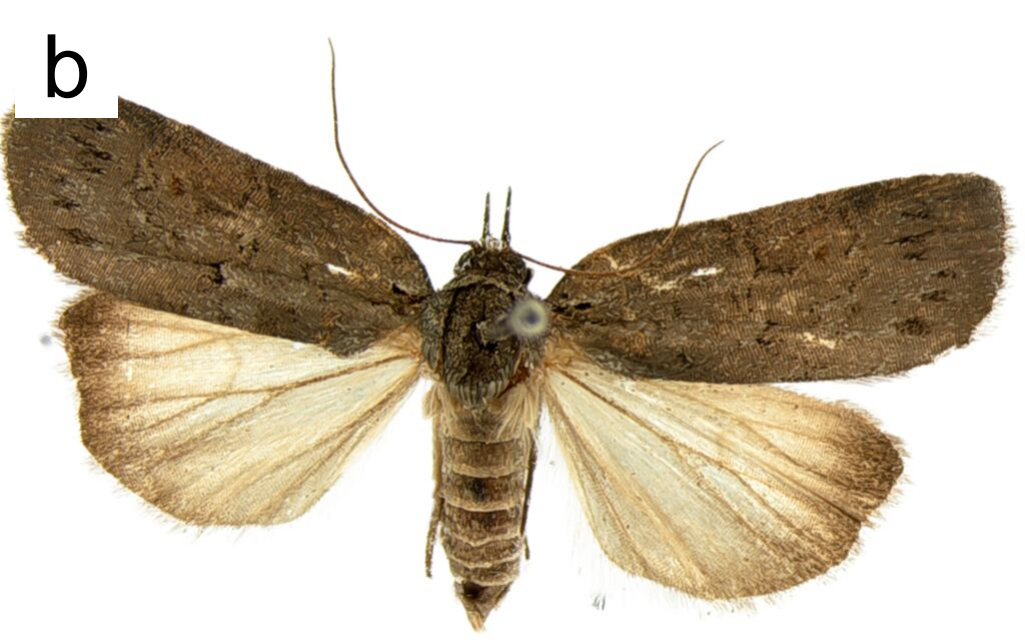
*N.asiatica*, female;

**Figure 1c.**
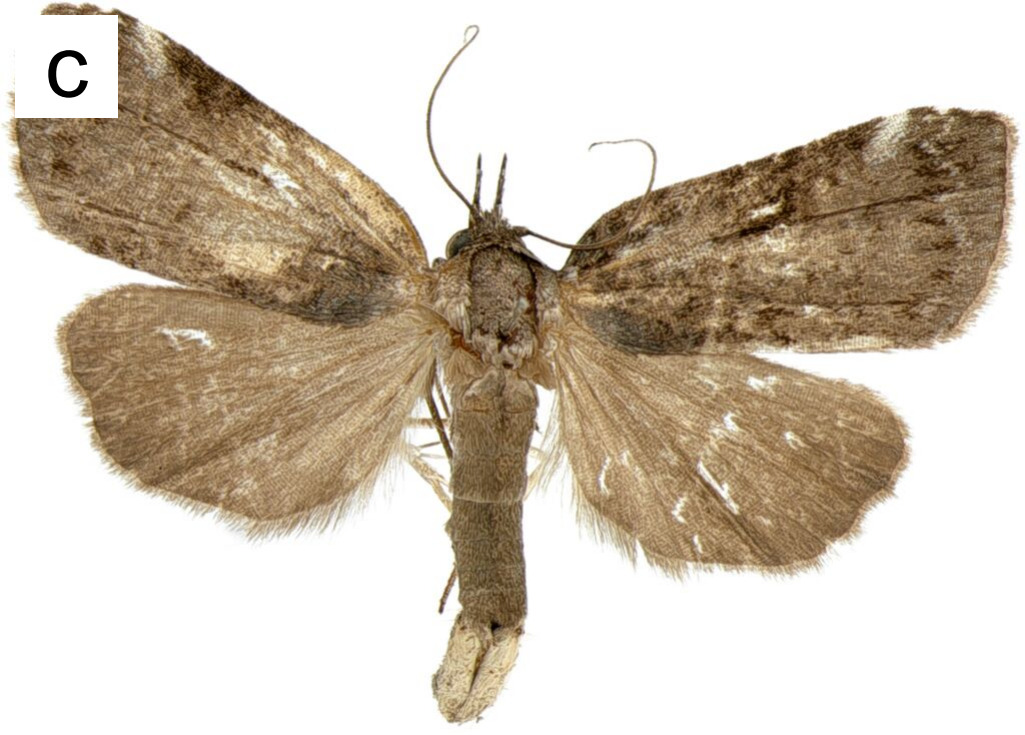
*N.coreana*, male;

**Figure 1d.**
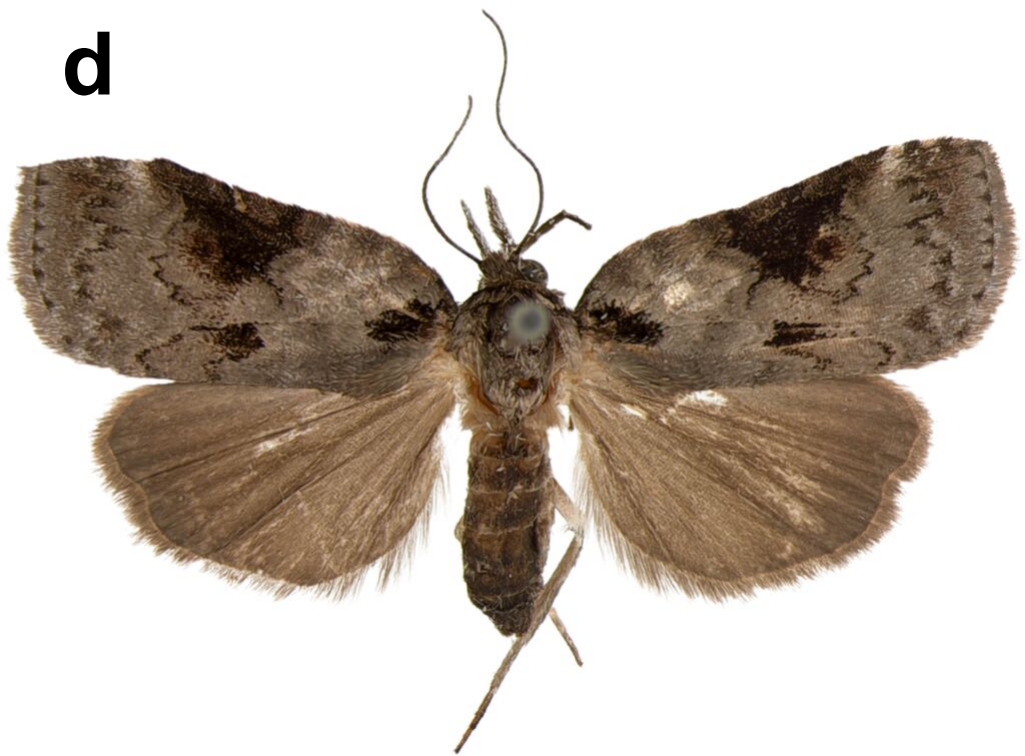
, female (Japanese sample).

**Figure 2a.**
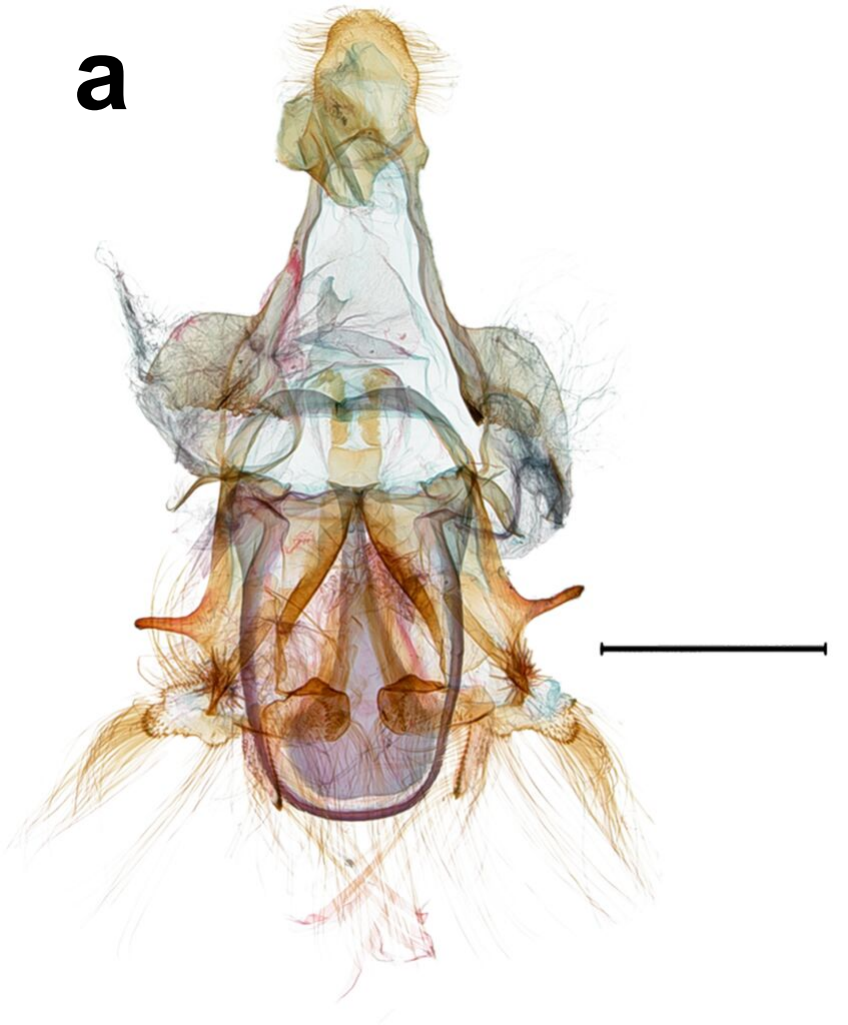
*N.asiatica* (INU-12086), genital capsule;

**Figure 2b.**
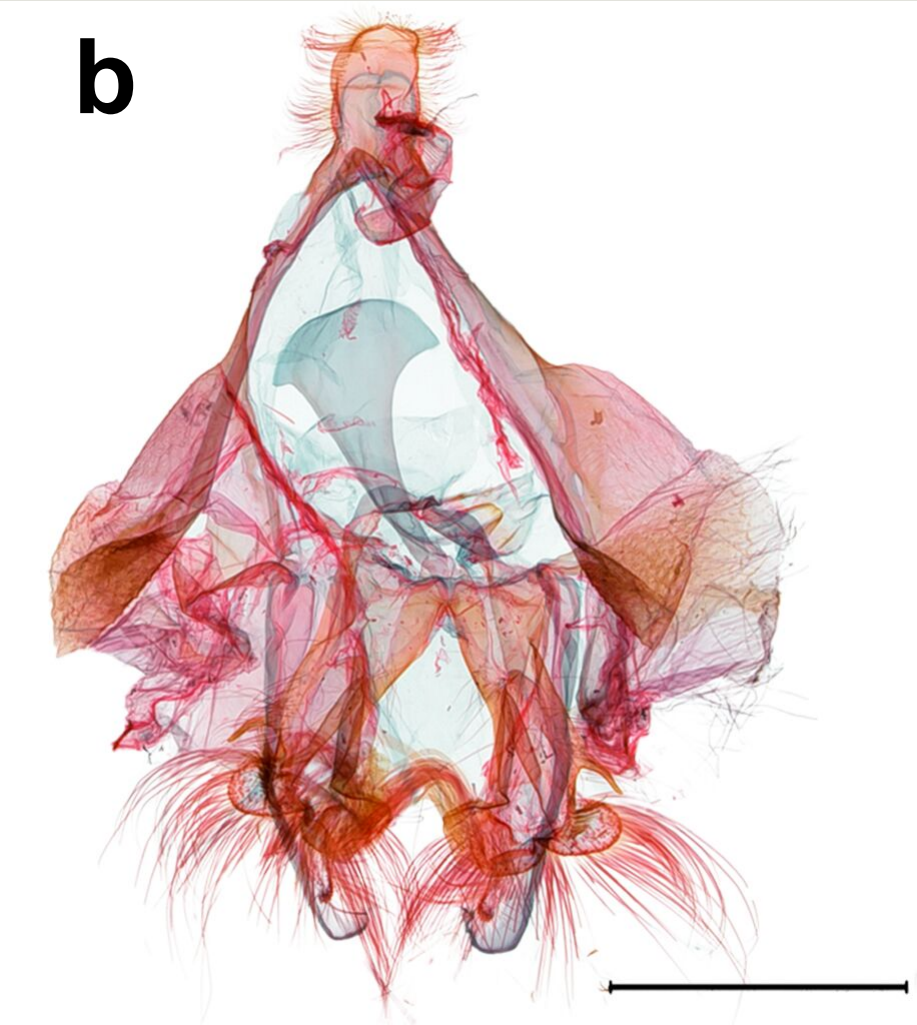
*N.coreana* (INU-12096), genital capsule;

**Figure 2c.**
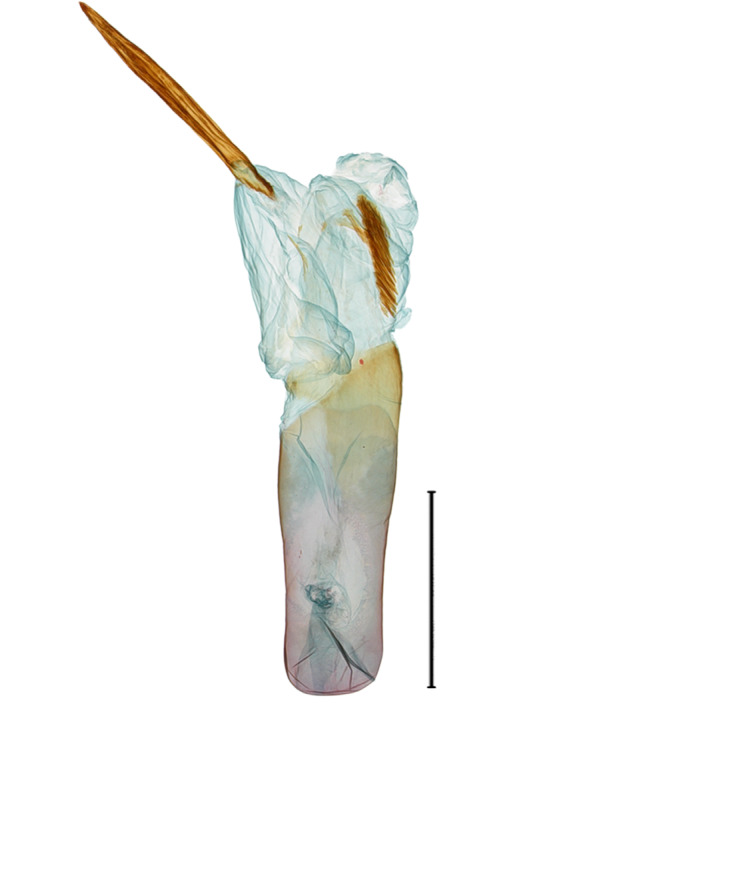
*N.asiatica*, aedeagus;

**Figure 2d.**
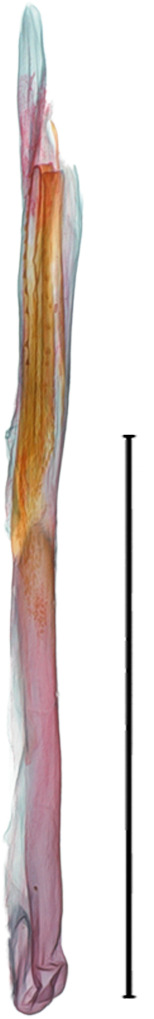
*N.coreana*, aedeagus;

**Figure 2e.**
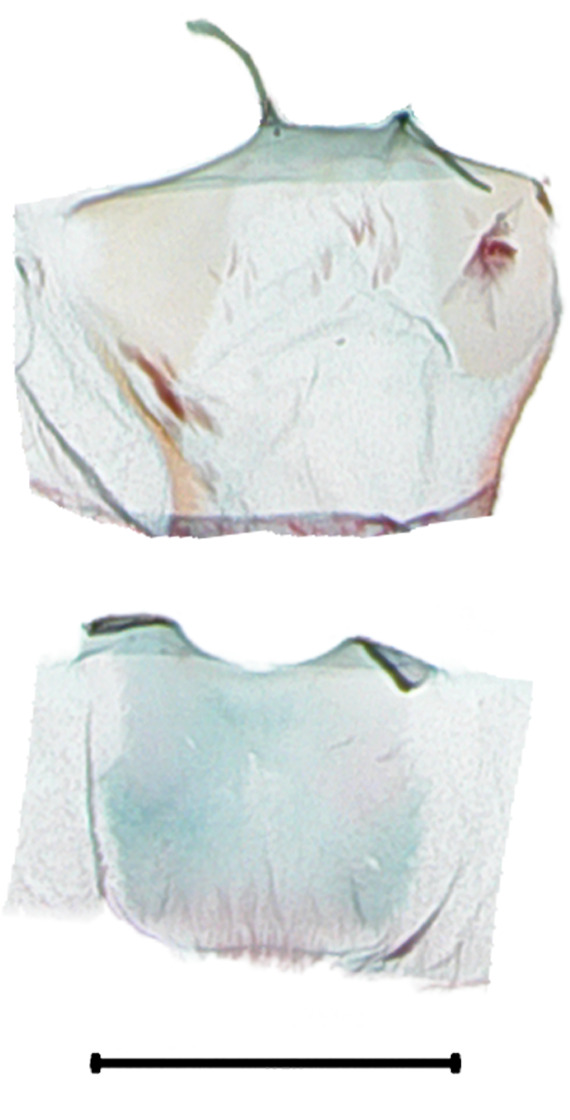
*N.asiatica*, 8^th^ segments;

**Figure 2f.**
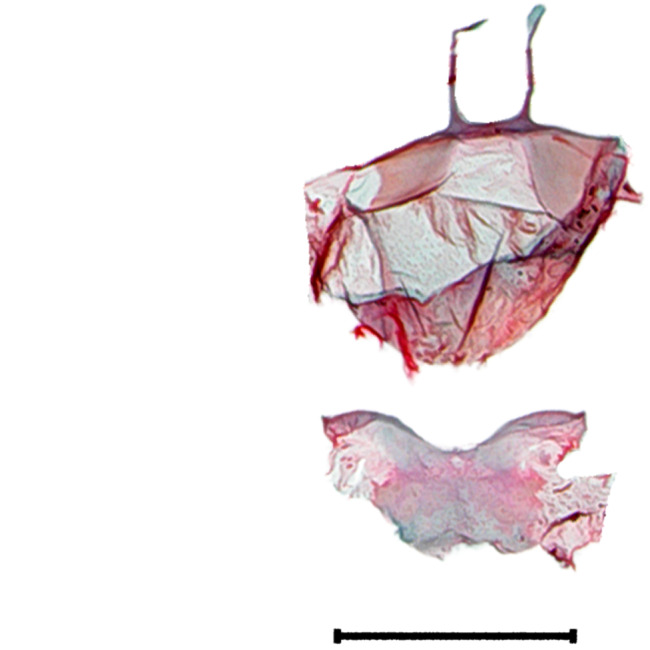
*N.coreana*, 8^th^ segments.

**Figure 3a.**
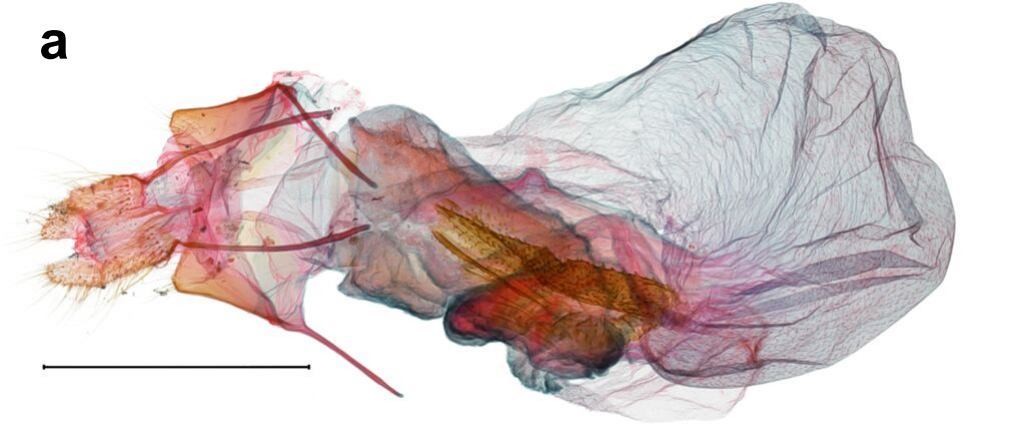
*N.asiatica* (INU-12085);

**Figure 3b.**
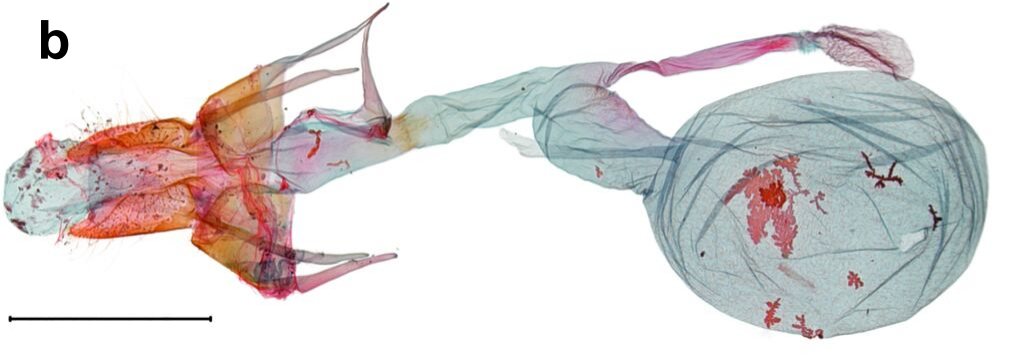
*N.coreana* (INU-11953, Japanese sample);

**Figure 3c.**
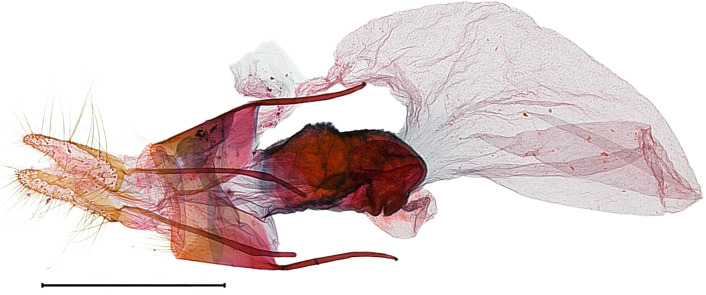
*N.degenerana* (INU-11844);

**Figure 3d.**
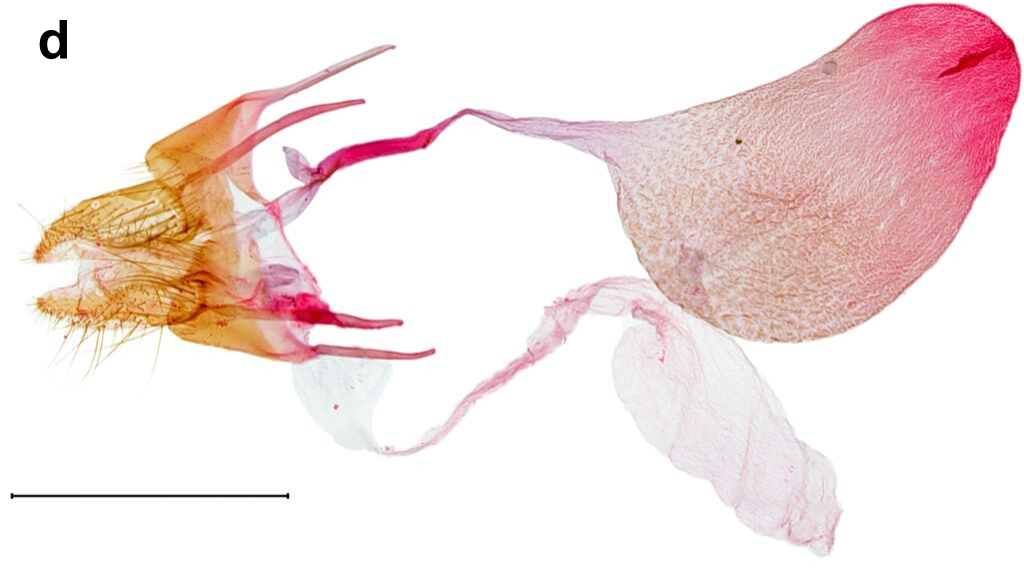
*N.dufayi* (INU-11973).

**Figure 4a.**
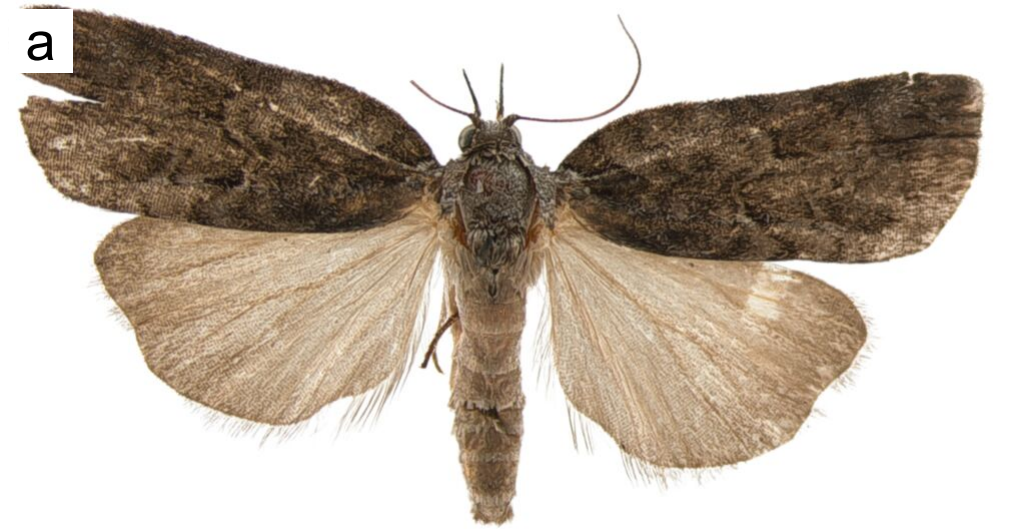
*N.degenerana*, male;

**Figure 4b.**
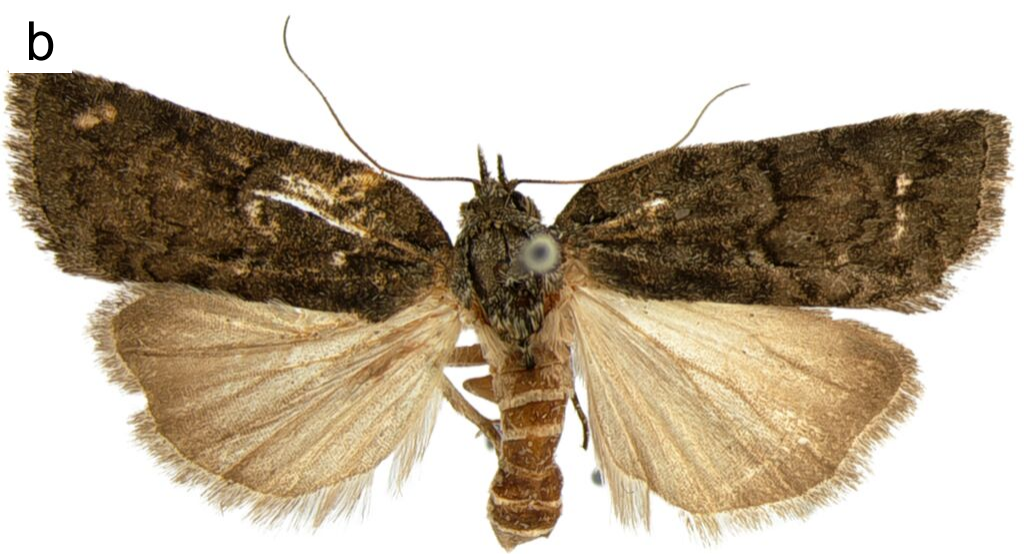
*N.degenerana*, female;

**Figure 4c.**
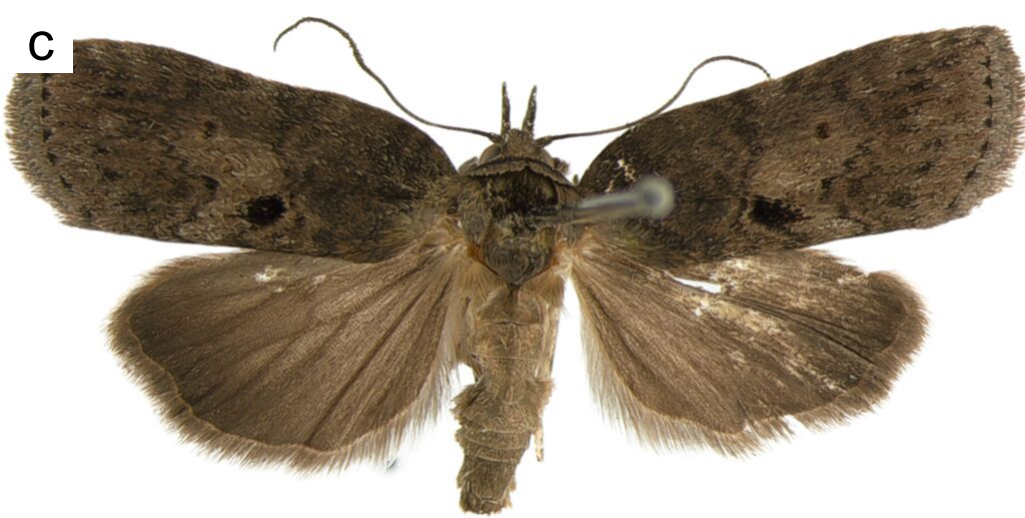
*N.dufayi*, male;

**Figure 4d.**
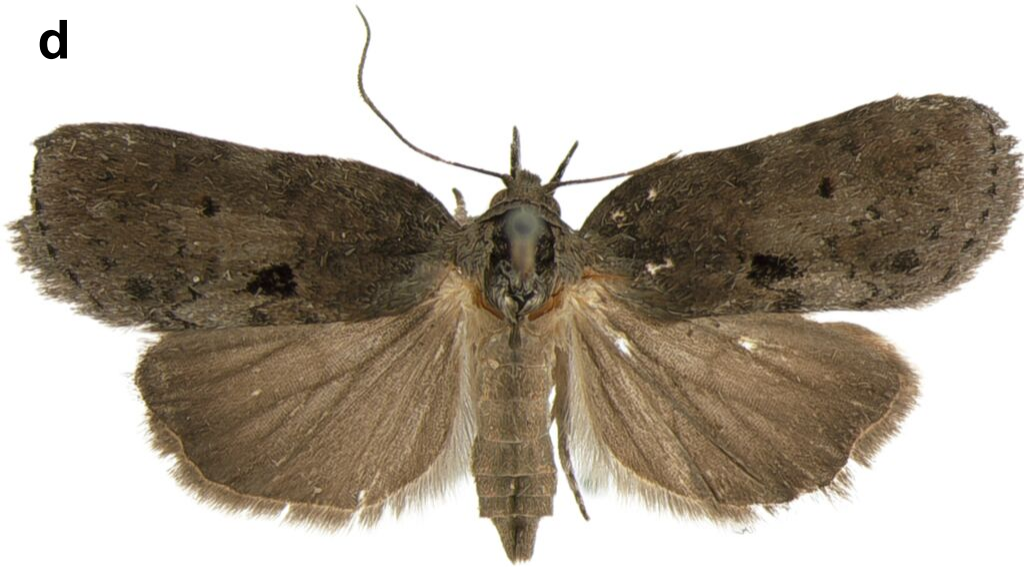
*N.dufayi*, female.

**Figure 5a.**
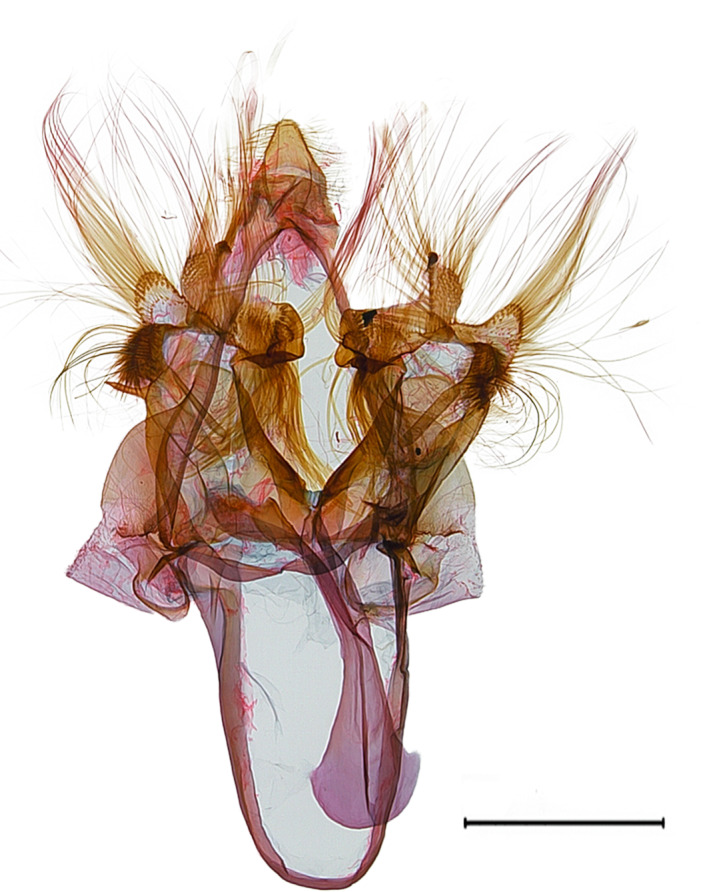
*N.degenerana* (INU-11609), genital capsule;

**Figure 5b.**
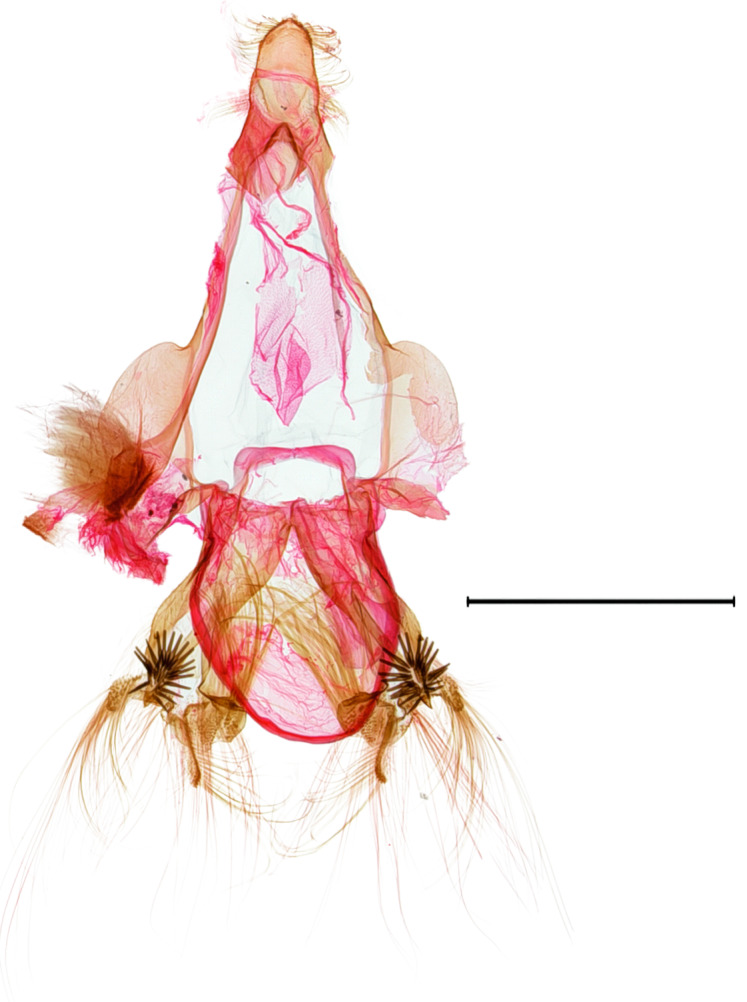
*N.dufayi* (INU-11972), genital capsule;

**Figure 5c.**
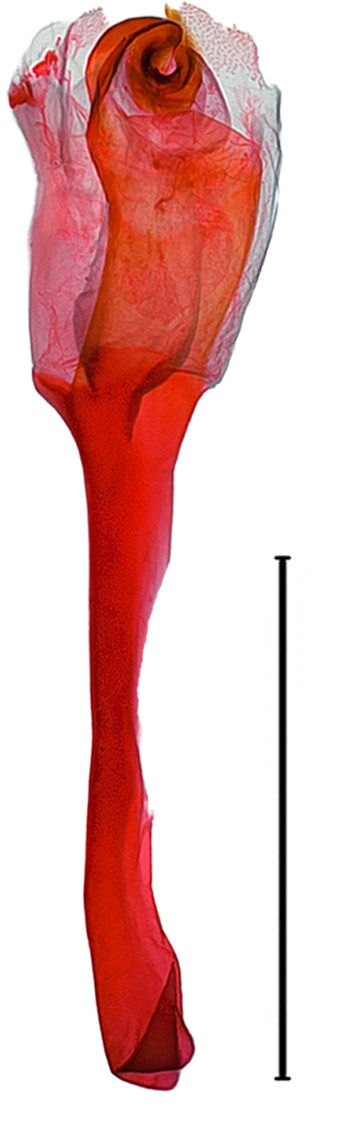
*N.degenerana*, aedeagus;

**Figure 5d.**
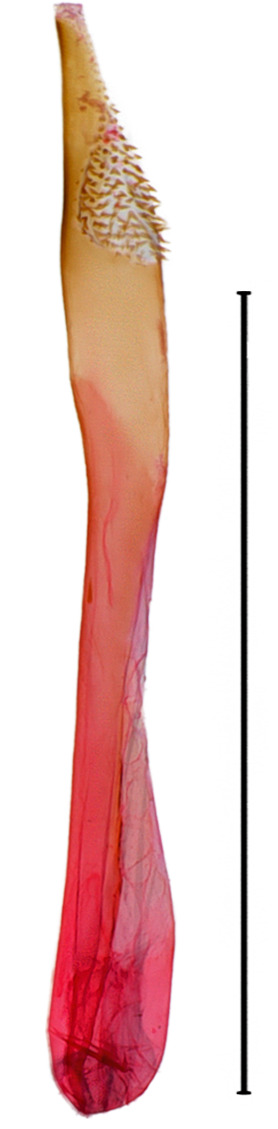
*N.dufayi* , aedeagus;

**Figure 5e.**
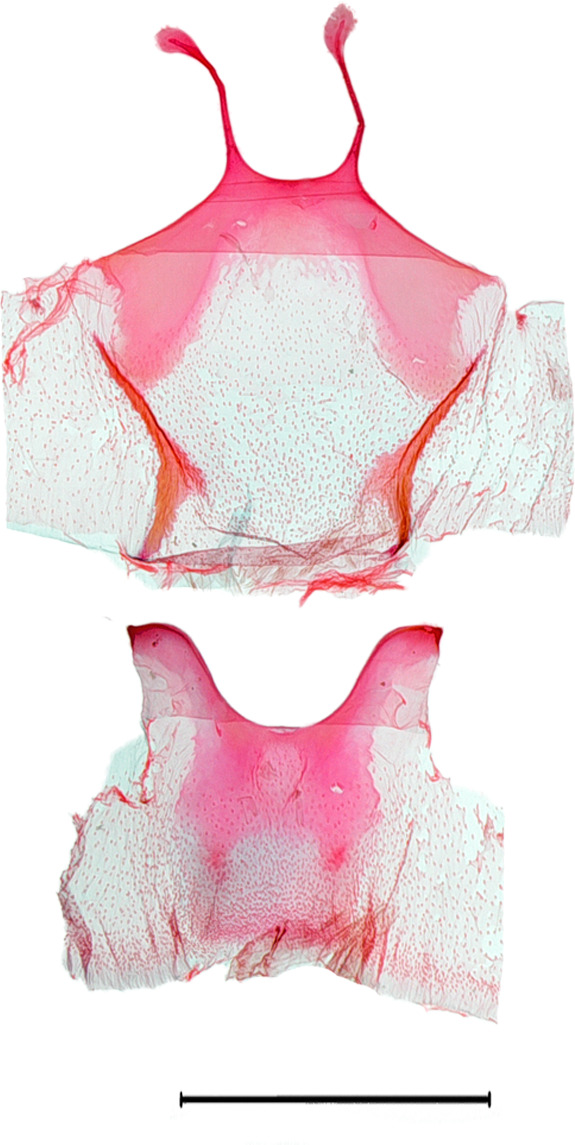
*N.degenerana*, 8^th^ segments;

**Figure 5f.**
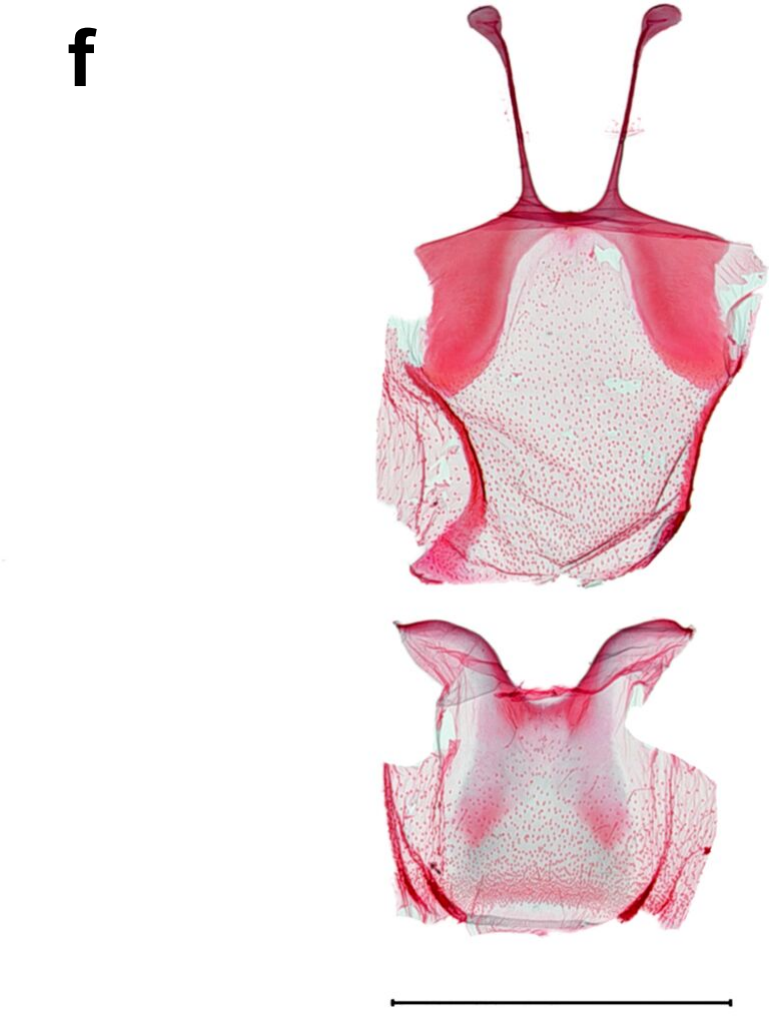
*N.dufayi*, 8^th^ segments.

**Figure 6a.**
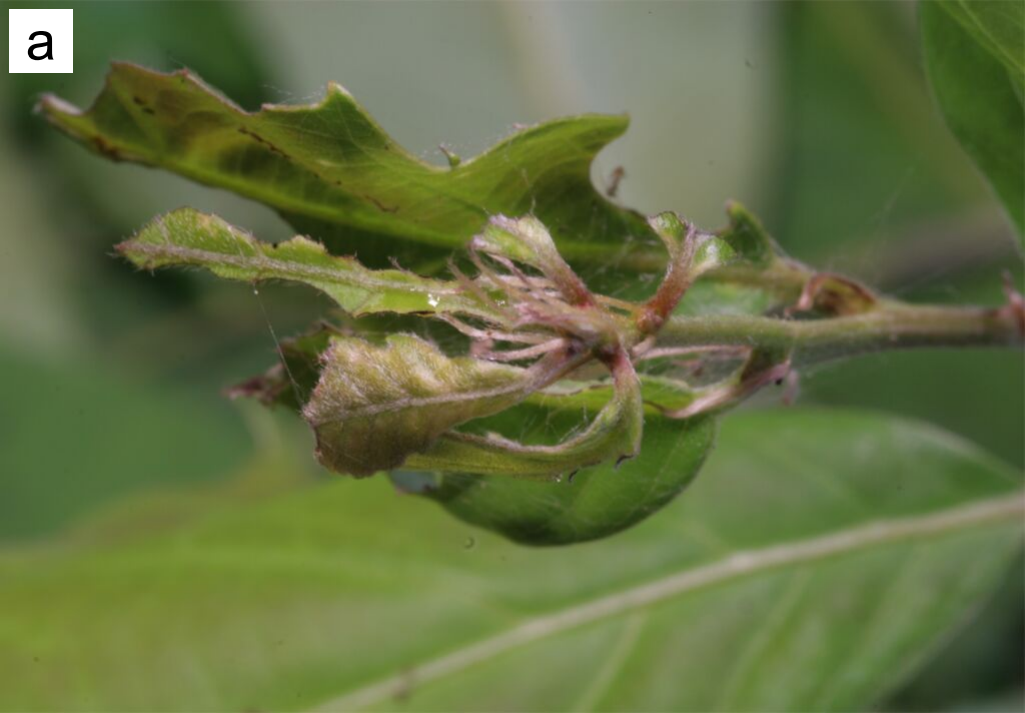
Host plant (*Quercusserrata*);

**Figure 6b.**
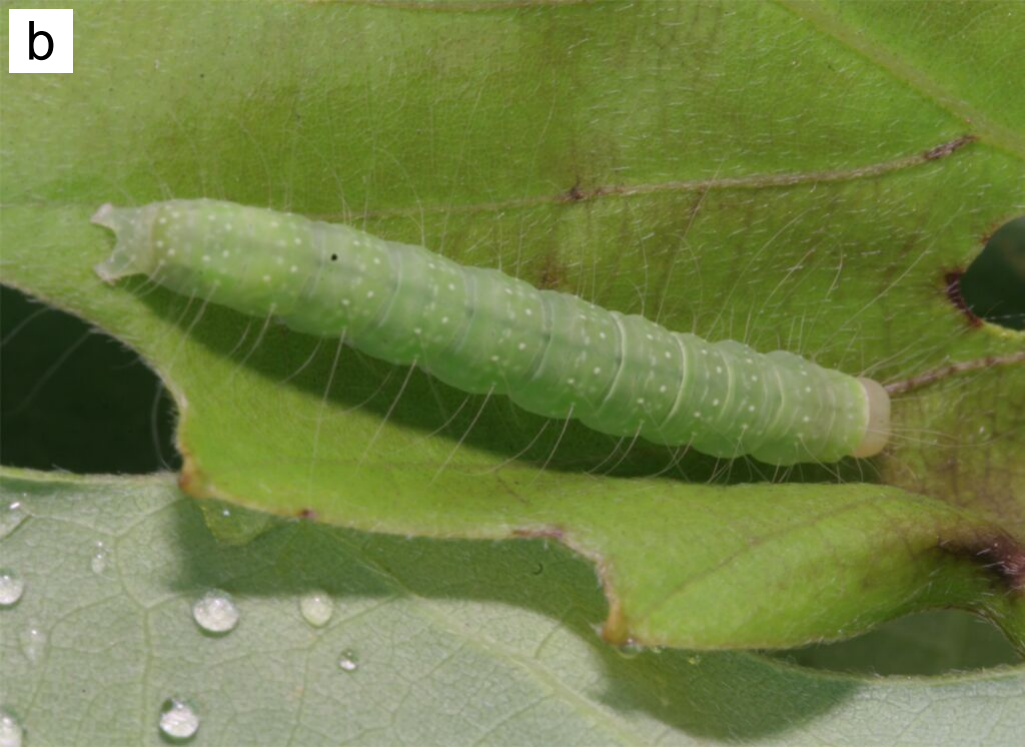
Final instar larva;

**Figure 6c.**
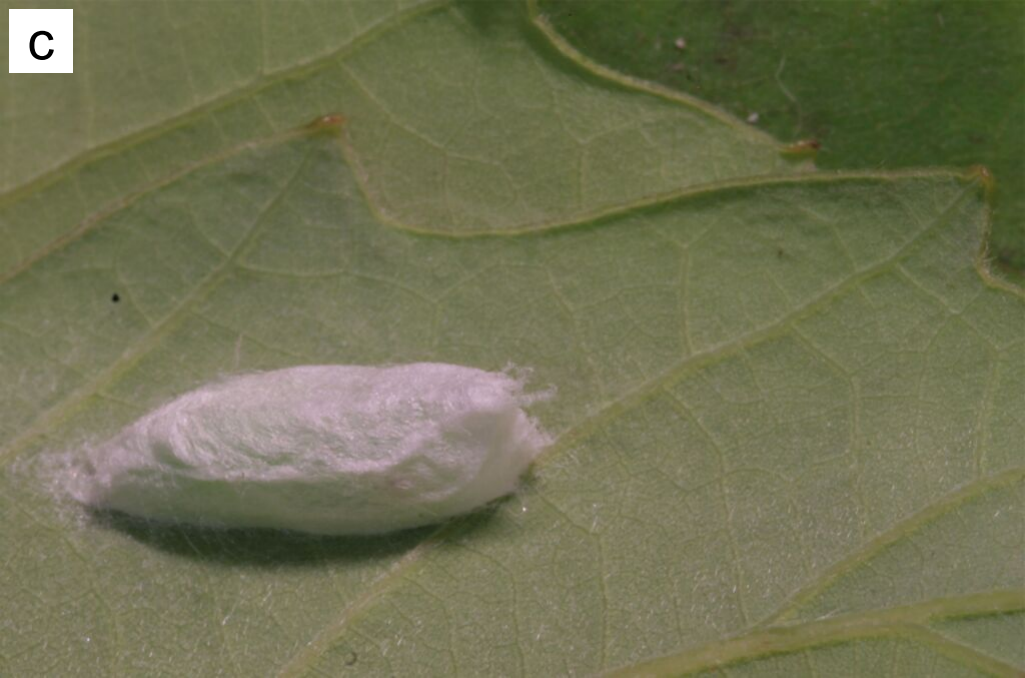
Cocoon;

**Figure 6d.**
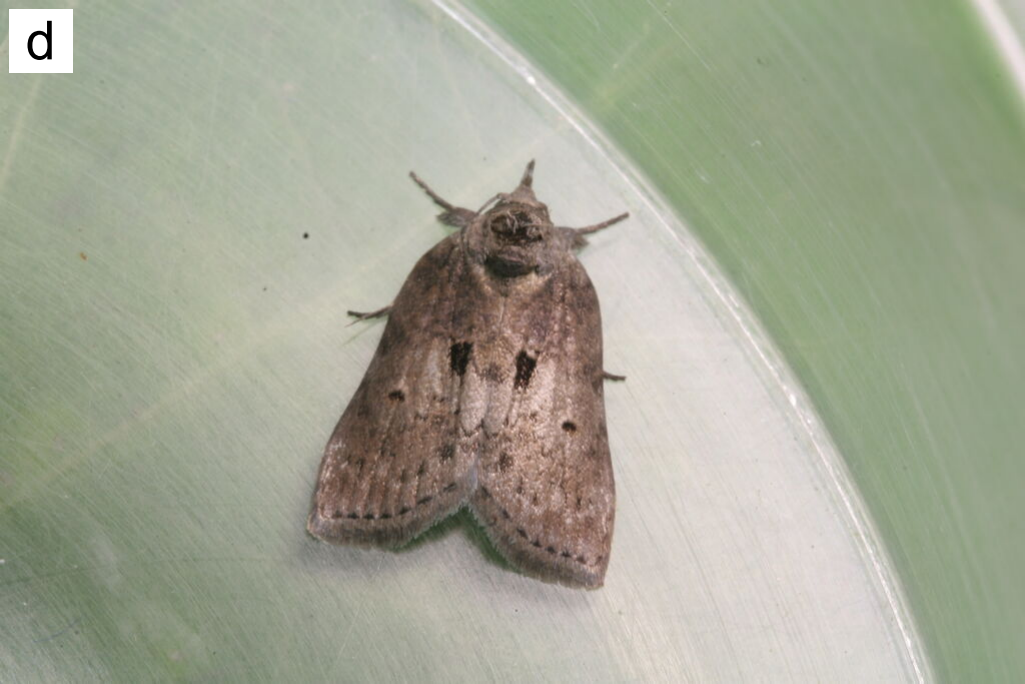
Newly hatched adult.
